# Psychometric Properties of a New Mexican Optimism Scale: Ethnopsychological Approach

**DOI:** 10.3390/ejihpe13120190

**Published:** 2023-11-25

**Authors:** Jorge Palacios-Delgado, Jessica Noemí Acevedo-Ibarra

**Affiliations:** 1 Unidad de Investigación en Neurociencias, Facultad de Psicología, Universidad del Valle de México, Campus Querétaro, Blvd. Juriquilla No. 1000 A Del. Santa Rosa Jáuregui, Querétaro 76230, Mexico; 2 Facultad de Psicología, Universidad del Valle de México, Campus Cumbres, Calle Av Las Palmas 5500, Cima de Las Cumbres, Monterrey 64610, Mexico

**Keywords:** optimism, scale, ethnopsychological approach

## Abstract

Background: In this paper, a Mexican Optimism Scale was constructed to investigate and estimate psychometric properties (reliability and validity based on the measurement model). The convergent validity and validity criterion were evaluated for a sample of young Mexicans. Methods: The scale was presented to 848 Mexican young people aged between 17 and 30 years from three different regions of Mexico. The scale was reduced to 20 items on the basis of CFA and analyses of internal consistency. Results: The confirmatory factor analysis (CFA) suggested a three-factor structure of optimism: affective resources, positive vision and hope. The data provided evidence for convergent validity with positive affect, negative affect and coping. Additionally, males scored higher on affective resources and positive vision than females. Finally, the results support the reliability of the instrument. Conclusions: The psychometric properties of the Mexican Optimism Scale proved to be highly acceptable and allow for a novel assessment of optimism from an ethnopsychological perspective. Validity, reliability and invariance were determined, as well as percentiles for the practical use of the scale. This scale may be of crucial importance for future research on optimism and health.

## 1. Introduction

In recent decades, positive psychology has positioned itself as a study perspective [1] that has demonstrated its effectiveness in interventions carried out under this approach [2,3,4]. One of the key constructs is optimism, which is defined as an emotional state associated with an expectation about the future that a person considers socially desirable [5].

Optimism is important in many areas of our lives [6] (Lyubomirsky, Sheldon & Schkade, 2005). It appears that people high in optimism are also more likely to engage in preventive health behaviors [7,8], including a low alcohol intake [9], in addition to having a lower risk of chronic diseases [10] and coping more effectively with stress and anxiety [11].

There are at least two ways to conceptualize and measure optimism. The most commonly accepted way is as a dispositional personality trait [12], with stable individual characteristics (expectations), where positive expectations of the future lead to goal achievement [13]. The second approach mentions that optimism is a self-regulation resource that serves to balance the relationship between negative and positive life events [14,15]. According to this perspective, people with a high degree of optimism generally tend to expect positive results [16]. People who are optimistic have a relatively positive set of feelings, even when things are difficult [10].

### 1.1. Measuring Optimism

In recent decades, international research on optimism has reported various instruments to assess this construct. Two of the most widely used measures of optimism are the Life Orientation Test LOT [17] and its successor, the Life Orientation Test—Revised LOT-R [18]), and these evaluate individual differences in optimism, including a positive direction of optimism and a negative direction of pessimism, under a one-dimensional approach. Despite being two of the most widely used instruments to measure optimism, these tests have been the subject of a series of criticisms when used, one of which is their one-dimensional structure, since evidence of a two-factor structure has been provided [19].

### 1.2. Alternative Optimism Scales

Research on optimism has developed alternative measures to assess this construct, such as the Questionnaire of Personal Optimism and Extended Social Optimism, POSO-E [20], which measures personal optimism, social optimism and self-efficacy. Despite measuring optimism in a comprehensive way, it is questionable whether it incorporates self-efficacy into the measurement of optimism, so it could lack validity, in addition to the fact that it has been found to be complex and difficult to interpret [21]. There is an instrument named State Optimism Measurement, SOM [22], which measures optimism as a state on a seven-item unidimensional scale, and it has construct validity and internal consistency between 0.92 and 0.96.

In Spain and Latin America, there are a few instruments in Spanish to assess optimism; one of these measures is the scale of *Optimismo/Pesimismo Disposicional-EOP* [23], created to discriminate between dispositional optimism and unrealistic optimism with 21 items, and it has content validity and a reliability of 0.85. The *Cuestionario de Optimismo* (COP) [24], which is a unidimensional scale of nine items, has construct validity, a relationship with personality and emotional intelligence and a reliability of 0.84. Finally, in the Mexican context, the IOS-G [25] attempts to measure optimism on a self-report scale of four positive items and four negative items. It has construct, convergent and discriminant validity, as well as an omega reliability of 0.86 and a Cronbach’s alpha of 0.85.

The instruments that attempt to measure optimism have important limitations, such as a one-dimensional structure [18,22,25] with a reduced representativeness of the construct, which limits the stability of the vision of the future in various life situations [17,24,25], in addition to having a low internal consistency ranging between 0.63 and 0.70 [17,26]. Additionally, in a study carried out on the Mexican population, a Cronbach’s alpha of 0.57 was obtained with the LOT-R scale [27].

In addition to these limitations, the previous scales incorporate Likert response options that range from agreement to disagreement to assess the temporality of the state of optimism [22,24,25], which may result in response biases (optimism items are worded positively, and pessimism items are worded negatively) [28]. Previous optimism scales present various criticisms and limitations, which justifies the need to create a different scale by reducing the previously identified limitations.

### 1.3. A New Mexican Optimism Scale

The measurement scale that we propose in this research is based on an ethnopsychological perspective [29,30]. Ethnopsychology studies universal and indigenous traits (psychologically relevant to each culture) and their consequences on the cognition and psychosocial behavior of individuals and groups within a human ecosystem [31]. This approach can shed light on the differences in and similarities of universal traits or idiosyncratic traits between cultural perspectives [30,32]. Under this approach, autochthonous (indigenous) traits have been found that are cardinal to the personality of the individual and typical of each culture [33,34]. Within the Mexican culture, there are socio-expressive and expressive-affiliative traits [35,36], with high levels of affection [37,38] and kindness [39], which allow certain opportunities to be obtained [40]. The development of the optimism measurement scale from an ethnopsychological perspective provides a contextualized view of how culture influences the way in which people interpret and express their optimism. Furthermore, this approach allows us to examine how culture can shape the way people maintain a positive outlook on life [32].

The second principle on which our scale proposal is based is the assumption that emotional states are integrated into affective resources through regulation strategies [41]. When a person uses strategies such as positive reappraisal, positive emotions can be generated [42], and positive emotions can expand an individual’s affective repertoire and build personal resources [43]; therefore, optimistic people experience more positive affect, even when things are tough [10,44].

Our instrument is different from the instruments previously developed [20,22,24,25], which have their roots in expectation value theory [17]. For Carver [14], optimism can be characterized as a cognitive construct but with emotional elements (whether good or bad things are expected to happen) and motivational implications (expectation levels). For us, optimism starts with a repertoire of positive emotional states that integrate affective resources that are used in the face of the different challenges that occur in life, so the conceptual basis of our scale is based on this foundation.

A second relevant contrast between our instrument and the instruments previously used is that, for example, the LOT and LOT-R were developed in an individualistic culture [45]; therefore, their measurements are oriented toward the expectations of results and the scope of achievements. Under this model [17,18], people are considered to remain committed to efforts to overcome adversity to achieve goals, as long as their expectations of success are sufficiently favorable. Our instrument is developed in a collectivist culture [45], where emotional and interpersonal relationships with the belonging group are encouraged [46]. Culture has a central effect on the development of instruments; thus, to measure optimism, specific patterns of individualistic cultures must be taken into consideration, which may be inappropriate for other cultures [47]. The findings shown stimulate the development of evaluation instruments and emphasize the importance of measuring optimism under a different theoretical perspective.

The creation of an alternative scale of optimism in young Mexicans is of interest because research has shown that it has an impact on health and has potential relevance as a predictor, mediator and protector of mental health during youth [48]. It is relevant to evaluate the psychometric properties of a new instrument in young people, as, in subsequent research, it will be possible to study the relationship between optimism and various risk behaviors [49,50,51]. Measuring optimism on a new theoretical basis will allow researchers to assess the effects of optimism interventions and their association with health outcomes that facilitate behavior modification [27]. Faced with this situation, in the present investigation, we consider the creation of a unique, valid and reliable measurement scale to evaluate optimism from an ethnopsychological perspective. 

### 1.4. Gender Differences in Optimism

There are studies that show differences between men and women in optimism. The evidence found [52,53,54,55] points out that men have a higher score on optimism than women; likewise, optimism, depending on age and maturity, occurs more frequently in women than in men [56,57]. Recently, it has been documented that females report experiencing both negative and positive affect more often than males, with gender differences being the greatest in negative emotions [58]. Moreover, this indicates that women experience greater emotionality than men [59]. In the cultural context of Mexico [35,39], emotional expression is socioculturally reinforced [60], and the characteristics of men are linked to the masculine role (autonomous, achievement-oriented and provider), whereas the characteristics of women are linked to the feminine role (sensitive, loving and affectionate). The above seems to indicate that men and women have different trajectories of life; therefore, visions about the future can be affected by the emotional experience and adversities that may arise in the cultural context in which an individual lives.

### 1.5. Objectives and Hypothesis

The new optimism scale has useful future implications for research and clinical practice; therefore, the objectives of this study are as follows: first, to estimate the psychometric properties (reliability and validity based on the internal structure) of a new instrument to measure optimism; second, to obtain convergent validity through the relationship with other variables; third, to evaluate the validity referring to a criterion, estimating the effect in men and women who present this characteristic; and, fourth, to obtain percentile scores in a sample of young Mexicans.

We hypothesize the following: (1) the new instrument will be represented in a multidimensional way, (2) it will have a positive correlation with coping as well as with positive emotional affect, (3) it will be negatively associated with negative affect, and (4) men will have higher scores than women.

## 2. Materials and Methods

### 2.1. Participants 

The sample consisted of 848 young people (51.7% female and 48.3% male), with an age range between 17 and 30 years (M = 20.44; SD = 2.6), selected via non-probabilistic sampling. The participants were from different cities in Mexico, clustered within three regions of the country, with 71% from the northern region, 25.4% from the central region and 3.7% from the southern region. In our sample of participants, 92.3% reported being single, 3.8% reported being married and 3.9% reported living in a free union. Of the participants, 59.9% studied, 10% worked and 30.1% studied and worked. These three regions of Mexico were selected to incorporate cultural diversity considering the living conditions, opportunities and challenges faced by young people in these regions of the country. 

### 2.2. Measures

#### 2.2.1. Mexican Optimism Scale (MOS)

Optimism can be conceived in different ways. We refer to optimism as an integrated emotional repertoire of personal resources that make it easier to visualize, respond to and buffer life’s circumstances in a positive way.

A specific instrument was developed for this study, adhering to the following steps: First, a scale was created from an ethnopsychological perspective [31,35]. Second, positive emotional states were incorporated [43]. Third, responses from an exploratory study were integrated [61]. Fourth, the inclusion of items that refer to optimism was avoided (e.g., I am an optimistic person). Fifth, a response format that allows for the frequency of each item to be measured was used. Sixth, universal indicators (*Etic*) were incorporated that are relevant, adequate and sensitive to the specific idiosyncratic characteristics (*Emic*) of the culture in which the construct is to be measured and operationalized [38,39,62]. 

The final instrument consists of 36 items with Likert-type response options. Each item presents a rating scale from 1 to 5 (never, few times, sometimes, many times and almost always). To obtain the total score, the item scores corresponding to the items about optimism are added up. A higher score represents being more optimistic.

In order to measure the convergent validity of the scale, we verified the associations with two constructs already used in previous studies: positive and negative affect [22] and coping [63].

#### 2.2.2. Positive and Negative Affect (PANAS)

The positive and negative affect scale (PANAS) is a 20-item self-report measure that assesses the frequency of positive and negative emotional experiences. Individuals rate how frequently they experience each emotional state on a 5-point Likert scale from 1 = not at all to 5 = extremely. The Spanish version was used [64]. The mean score of positive and negative affect was computed. The internal consistency reliability in the present sample was α = 0.89 (CI 95% = 0.88–0.90) for the positive subscale and α = 0.86 for the negative subscale (CI 95% = 0.84- 0.87).

#### 2.2.3. Coping Strategies (CSI-SF)

Adapted to Spanish [65], CSI-SF consists of 16 items on a Likert scale (ranging from 1 = never to 5 = always), and it evaluates strategies, focusing on commitment and avoidance strategies, and two categories of coping, problem solving and resolution through emotion. The reliability for this study’s Cronbach’s alpha (α) was problem-focused engagement (PFE) = 0.59 (CI95% = 0.55–0.64), problem-focused disengagement (PFD) = 0.65 (CI95% = 0.61–0.68), emotion-focused engagement (EFE) = 0.83 (CI 95% = 0.81–0.85) and emotion-focused disengagement (EFD) = 0.67 (CI95% = 0.63–0.71).

### 2.3. Procedure 

The information was obtained over two months. The instrument was administered to the participants digitally through a form developed in Google Forms, and it was shared through social networks, with an approximate response time of 20 min. The purpose of the study was explained on the form, and the participants were asked to answer honestly, as their responses would be used for research.

### 2.4. Statistical Analysis

Statistical analyses were conducted using JASP Program version 0.9.2 [66]. First, distributions and item discrimination were reviewed. Univariate and multivariate normality tests were performed for the total sample, applying the Kolmogorov–Smirnov test and skewness and kurtosis tests. Moreover, psychometric data were used as item–total correlation criteria, removing those that obtained a correlation lower than 0.30. The results obtained report the suitability of the items. 

To continue with the analysis, participants were randomly split into two equivalent subsamples. To evaluate the psychometric characteristics of the instrument, the construct validity was examined in two stages: At its exploratory level, an exploratory factor analysis (EFA) was applied in the first subsample (*n* = 400). Sample adequacy measures were examined, and the Kaiser–Meyer–Olkin (*KMO* ≥ 0.80) and the Bartlett sphericity tests (*p* < 0.05) were performed; the estimation method was the maximum likelihood, with Oblimin rotation.

In the second subsample (*n* = 448), a confirmatory factor analysis (CFA) was applied through the maximum likelihood estimation approach. To evaluate its goodness of fit, we computed the chi-square (χ^2^), root-mean-square error of approximation (RMSEA), comparative fit index (CFI), relative fit index (RFI), incremental fit index (IFI), normed fit index (NFI) and standardized root mean residual (SRMR). To evaluate the cut-off and determine the model fit, we followed the guidelines published by the *European Journal of Psychological Assessment* [67]. The internal consistency was measured using the Cronbach’s alpha and McDonald’s omega of the resulting factors in the scale. 

To obtain further information about the construct validity of the scale, a convergent validity analysis (average variance extracted: AVE) was performed. For AVE values, >0.50 is needed. The discriminant validity was evaluated by using the cross-loading of an indicator, the Fornell and Larcker criterion was considered. Subsequently, a CFA was carried out to test for the measurement invariance using data from the entire sample. Measurement invariance across genders was tested, and the criteria used for the final decision were Δ*CFI* ≤ 0.01 and Δ*RMSEA* ≤ 0.015 [68]. To determine discriminant validity, *Spearman’s Rho* correlations between the scores obtained with the optimism scale and those obtained with the PANAS and coping scale were carried out. *Mann–Whitney U* tests were used to determine the differences in optimism between men and women, with a significance level of *p* > 0.05. Effect sizes were reported as *r* [*r = z/√n*] for non-parametric data, and they were interpreted using the conventional metrics small = 0.10, medium = 0.30 and large = 0.50 [69]. Finally, logistic regression models were performed to determine the ratio of optimism in men and women. 

## 3. Results

### 3.1. Descriptive Statistics 

Table 1 presents the descriptive statistics of the individual items of the scale. For skewness and kurtosis, we found that many of the items fell within the ±1.5 range [70], whereas the other items had higher values. Nonetheless, the Kolmogorov–Smirnov normality tests showed that all items were distributed in a non-normal way (*p* < 0.01).

### 3.2. Exploratory Factor Analysis 

The EFA was performed in the first sample. The sample adequacy index was evaluated (KMO = 0.976; Bartlett: *X^2^* = 11,149.898; *gl* = 630; *p* < 0.001) and considered excellent, indicating the multidimensionality of the scale. The exploratory factorial analysis showed three factors with a factorial weight greater than 0.40 and percentages of explained variance of 27%, 15% and 14%, as well as conceptual clarity in the grouping of the items (Table 2). The criterion for selecting a factor was an eigenvalue greater than 1.0. 

### 3.3. Confirmatory Factor Analysis 

**Figure 1:**
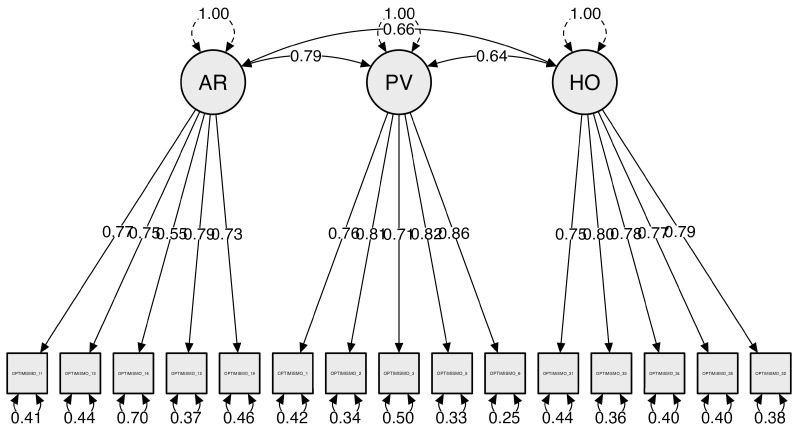
Confirmatory analysis of optimism scale (loadings were standardized).

Second, to confirm the factorial structure obtained in the EFA, a model with a CFA was tested on the second sample. The estimation used was the maximum likelihood with the robust method [71]. Three models were tested, namely, Model 1, a model based on the AFE results and the CFA model with three factors, considering the modification indices to find the appropriate fit on the scale. The modification indices were considered to find the best fit with respect to the EFA. Adjustments were made by eliminating the elements that did not conform to the established model. Considering the correlations between PV and AR, a two-factor model was tested. Table 3 shows the results of the models. The CFA model with three factors showed an optimal fit to the data. The indicators of the absolute goodness of fit, incremental adjustment and parsimony adjustment were found to be adequate [72]. The scale provides an adequate explanation of optimism, with coefficients ranging from 0.54 to 0.79 (Figure 1). 

### 3.4. Convergent and Discriminant Validity 

The next step in the evaluation of the measurement model involved the assessment of the discriminant validity of the study construct. This analysis specifically showed the extent to which the construct of optimism was distinct from other constructs in the structural model. The AVEs were acceptable, explaining 54% of the variance of the indicators that compose the construct. 

The discriminant validity was assessed using the Fornell and Larcker criterion [73] by comparing the square root of each AVE in the diagonal with the correlation coefficients (off-diagonal) for each construct. Table 4 provides the results of this criterion in this study. The data refer to how empirically distinct one construct is from the other constructs in the structural model. It also implies that the discriminant validity can be accepted for this measurement model and supports the discriminant validity between the constructs. 

### 3.5. Reliability Analysis

The reliability, determined via internal consistency alpha and omega coefficients, was calculated using two methods. In the EFA, the F1 had an index of α = 0.95 (CI95% = 0.95–0.96), the F2 had a reliability of α = 0.90 (CI95% = 0.91–0.92), and the F3 had an index of α = 0.90 (CI95% = 0.89–0.91). 

In the CFA, good values were found for the positive vision factor (α = 0.88 (CI95% = 0.87–0.89); ω = 0.88 (CI95% = 0.87–0.90)), affective resources (α = 0.83 (CI95% = 0.82–0.85); ω = 0.84 (CI95% = 0.82–0.85)) and hope (α = 0.88 (CI95% = 0.87–0.89); ω = 0.88 (CI95% = 0.87–0.89)).

### 3.6. Invariance Analysis

The measurement invariance across sex was determined. The configural invariance (M1), metric invariance (M2), scalar invariance (M3) and strict invariance (M4) were progressively calculated. The results are presented in Table 5. The invariance tests indicated that the model had an acceptable fit for both the male and female samples. A good adjustment of M1 was observed, with similar values for M2, with minimal differences for M3 and M4. The constraints imposed on the model revealed the invariance of the factor loadings.

### 3.7. Criterion and Concurrent Validity

In order to obtain the criterion validity, each optimism factor was compared between men and women. The data showed a statistically significant difference in two factors of optimism between men and women, with higher scores for men in positive vision (men: *M* = 28.32, *SD* = 6.1; women: *M* = 27.52, *SD* = 5.8; *U* = 82,775.500; *p* < 0.05; *r_B_* = 0.08) and affective resources (men: *M* = 27.00, *SD* = 6.0; women: *M* = 25.43, *SD* = 5.4; *U* = 74,468.000; *p* < 0.001; *r_B_* = 0.15). Both men and women scored similarly in hope (men: *M* = 15.23, *SD* = 3.2; women: *M* = 15.05, *SD* = 3.1; *U* = 85,709.000; *p* = 0.25; *r_B_* = 0.04). The logistic regression model was statistically significant (χ^2^ (844) = 23.300, *p* < 0.001). Men had a higher score in affective resources (odds ratio = 1.09; (CI95% = 1.05–1.14), with an *R*^2^ Nagelkerke of 0.03. 

The correlations of the optimism scale with the PANAS and coping scale (convergent validity) were analyzed (Table 6). The MOS demonstrated convergent validity, with positive and negative Spearman’s correlation coefficients with the hypothesized related constructs of optimism. The three factors of the MOS were the most highly correlated with positive affect and moderately correlated with negative affect.

When examining the other theoretically related yet distinct measures, the correlations were high, positive and significant with problem-focused engagement (PFE) coping and weaker but still positive with emotion-focused engagement (EFE). In addition, the correlations with emotion-focused disengagement (EFD) coping were low and negative, and there were no correlations with problem-focused engagement coping. These results provide sufficient evidence for convergent and discriminant validity.

### 3.8. Percentile Scores

Based on the distribution of the scores obtained from the total sample, to interpret the results of the scale, Table 7 shows the percentiles of the scores of each factor of optimism separated for men and women.

## 4. Discussion

The objective of this study was to create a new scale to measure optimism and to determine its psychometric properties. Optimism is defined in this paper as an integrated emotional repertoire of personal resources that facilitate visualizing, responding to and cushioning life’s circumstances in a positive way. From this perspective, a scale composed of a total of 20 items that converge separately on three factors was derived. The psychometric results obtained for the scale developed in this paper demonstrate the validity, reliability and cultural sensitivity of the optimism measure from an ethnopsychological perspective.

### 4.1. Theoretical Implications

First, the structure was assessed through exploratory and confirmatory factorial analyses. The CFA confirmed a three-factor structure. Based on the theoretical background and the results obtained from the CFA model, optimism is represented in the first factor by an expressive repertoire named affective resources. This factor is consistent with the evidence found on the expressive traits of Mexican people [35,38] and their display of affection [37,39]. The integration of this factor confirms our proposal of assuming that Mexican people display affective states that extend as positive emotional resources that serve to face the adversities of life. Previous evidence [10,12] pointed out that, in the face of an adverse situation, an individual can regulate their affect in a negative or positive way. The findings support the proposal that positive emotions extend into resources for people to take action [41]. Our results can be incorporated into self-regulation models to address behavioral problems [9,12] and other health threats [27].

The second factor measures a personal strength characterized by a positive perspective on life and adverse situations; this is called positive vision. The confirmation of this factor represents the central nucleus of the meaning of optimism. Through these items, we can identify the way in which people see life, finding the good side of each situation; at the same time, the items represent a positive way of dealing with difficult situations. Some studies [5,8,15] point out that optimists use strategies that involve seeing the positive in situations. It is important to note that optimistic people feel that life circumstances will improve, so they see life in a positive way, managing the challenges and adversities that come their way [12,22,24].

The third factor represents the longing for a positive future, which each individual assigns to external events; for this reason, this factor is named hope. These items constitute persistence in the face of adversity and adjustment from negative to positive changes in life. Optimists tend to see negative events as attributable to factors outside of themselves, which allows them to persist in the face of adversity [22]. 

Items that are grouped as hope are a form of positive and adaptive coping, where longing provides relief from negative conditions [74]. Although hope and optimism are related constructs, empirically, they may have different results [75,76]. We can distinguish our measure of optimism from hope because, traditionally, hope has three domains: the cognitive–temporal, affective–behavioral and affiliative–contextual domains [75]. The affiliative–contextual domain refers to the interdependence and interconnection between oneself, others and spirituality, with elements related to perceived social support [76], providing support to the hope factor that we included in our instrument. For us, hope is a connection with oneself or others, and it is linked to something external, such as family or spirituality, to face adversity.

The convergent validity analysis indicated a significant association with the three factors of the new optimism scale. Positive vision, affective resources and hope were positively and significantly related to the positive affect of the PANAS and negatively and significantly related to the negative affect of the PANAS. This result was also found in previous studies in other contexts [22,28,77]. The strong associations of the three factors of the new optimism scale with the PANAS, especially with positive affect, confirm that positive emotional states are extended as affective resources [41,43]. These findings also support the ethnopsychology perspective [31,38], with the Mexican affect [35,37,39] being supported by the correlations between the PANAS and the affective resources found in the present study. 

In addition, we would like to discuss the association of positive vision, affective resources and hope with coping. The analyses revealed positive and significant relationships with problem-focused engagement (PFE) coping and emotion-focused engagement (EFE) coping, in addition to negative and significant associations with emotion-focused avoidance (EFD) coping. Furthermore, affective resources and hope were negatively and significantly related to problem-focused avoidance (PFD) coping, although the relationships were weak. These results agree with those of other studies [63,78] showing that optimism is positively associated with engagement coping, which seeks to reduce or manage stressors or emotional consequences, and that it is negatively associated with avoidance coping, which seeks to withdraw from stressors. In addition, people who possess psychological resources such as proactive coping are capable of maintaining a positive vision in environments that are considered highly stressful [79]; therefore, optimism is related to more effective coping strategies [13,80]. The directions of each of these correlations were exactly as expected, and they offered initial support for confirming the discriminant validity of our optimism scale.

Further, we performed a multigroup confirmatory factor analysis to test whether the scale is invariant across sexes. The findings show that the factorial structure remained stable in both sexes; that is, men and women responded similarly to the scale, allowing for meaningful comparisons across genders. This suggests that the items measure optimism in a similar way in both groups, so it is possible to use the MOS in future comparative studies. Based on the scores of the three factors, the results suggest that the MOS can discriminate between subgroups based on sex. Mexican men scored higher than women in positive vision and affective resources, which is consistent with other studies [55,81,82,83] that indicate that men are more optimistic than women. The differences due to sex seem to indicate that men of this age group develop personal resources that they use to positively face adversities that arise. Our results may also be due to the fact that women experience greater and/or different stressors than men from adolescence, according to the cultural roles established for each sex [84].

From a cultural perspective, differences in optimism are explained by sociocultural characteristics [35,60]. Women focus on negative emotions and their consequences, that is, more so than men [85], whereas men focus on problem solving [86]. In the same way, other investigations report that men have higher scores on the emotional and mental health subscale of quality of life [84,87]. 

Regarding the analysis of internal consistency, the obtained Cronbach’s alpha and McDonald’s omega coefficients indicated an acceptable level of reliability. The reliability levels of our instrument are higher than those of previously used scales [17,26,55]. Our results are in line with the estimates of scales developed by other researchers in different cultural contexts [22,23,24], as well as those developed in the Mexican context [25]. 

When interpreting the results of the optimism scale, the percentiles make it easier to achieve adequate normative data for each country since they allow for a comparison of the scores of optimism obtained by an individual with respect to the reference group. However, it is important to consider the variations in the culture in which the scale is applied, as sociocultural differences influence the result [39].

### 4.2. Limitations and Suggestions

One potential limitation of our research is the use of convenience samples since they only represent a subsample of young people from Mexico, restricting the generalizability of the results. In future studies, it will be necessary to apply the construct to different types of samples, such as teenagers and adults, in order to corroborate the reliability and theoretical structure of the scale obtained. Before generalizing the results to other Latin American countries, it is necessary to obtain more empirical evidence and to establish normalized values for different countries, including European countries, since the scores may differ. Therefore, future studies will be aimed at obtaining cross-cultural validity of the instrument and confirming the findings.

Another limitation is that the responses might be biased because of social desirability, which is a common and known issue of self-report measures; however, this effect is likely to be reduced when collecting data online and anonymously. Furthermore, it is important to establish the divergent validity of optimism with regard to related constructs from positive psychology, such as gratitude, well-being and kindness. Comparisons of the Mexican Optimism Scale with well-established measures of optimism, such as SOM [22] or COP [24], would allow for the testing of concurrent validity.

### 4.3. Contributions and Strengths 

On the basis of our data, we would like to discuss the limitations and contributions to ethnopsychology. Ethnopsychology is a valuable approach to understanding the interaction between culture and its characteristic features [30]; therefore, it is essential to recognize its possibilities and limitations when designing measurement instruments in this field. Ethnopsychology challenges the idea that certain psychological characteristics are universal, contributing to a more complete view of human diversity [31,32].

The findings of ethnopsychology may be specific to a culture or group, which makes generalization to other populations difficult [32,33]; therefore, it is important to be cautious when extrapolating the results obtained from our measurement scale to other cultures. Given the complexity of developing indigenous measures (such as the scale that we developed), it is helpful to use an ethnopsychological approach and ensure that both the *etic* and *emic* perspectives are represented in that approach. In this scenario, we seek to provide evidence on the *etic* and *emic* dilemma [62] in the development of measuring instruments for optimism. Sticking to the *emic* proposal, first, our study provides evidence showing that, although optimism is a universal trait, our scale demonstrates that it can be culturally idiosyncratic. Second, universal traits can manifest in different ways across cultures. Third, the new measure of optimism in this research is a valid, reliable and culturally relevant measure.

The contribution of this research is the creation of a new scale and empirical estimations of different aspects of validity to measure optimism through three factors in a valid, reliable and culturally sensitive way in young Mexican people. One of the strengths of the scale is the theoretical perspective on which it is based (ethnopsychology and positive psychology). In the findings, the expression of the positive affect typical of Mexican culture stands out as a resource used to face adversity. This strength allows us to differentiate our instrument from other scales that are based on expectations of achieving a goal, a characteristic behavior of individualistic cultures. We highlight as a second strength that the developed scale incorporates affect regulation instead of stable attributes of the personality structure. A third strength is in the response options, which make it possible to quantify the frequency with which affective or visionary resources are used, unlike in previous scales that use an attitudinal assessment to assess the temporality of optimism [22,24,25].

Before concluding, we would like to mention that future studies will allow us to determine the effect that optimism has on behavioral economics (heuristics and biases). In addition, our future research is heading in two directions: the first is to test models that determine the effect of optimism on mental health, and the second is to carry out interventions based on the evidence obtained that have an impact on increasing optimism. It has been observed that optimistic youth experience positive emotions, are more engaged in their daily activities, have more supportive relationships and have a better sense of direction in life [88]. Therefore, optimism should be integrated into prevention and intervention programs for mental and physical health to improve well-being [80].

### 4.4. Practical Implications

Positive psychology is a relatively new field, and researchers have developed measurement instruments so that psychology professionals can use them in their research, in clinical practice or in different interventions. The new scale created for this research can be incorporated into psychological interventions focused on positive psychology, which use optimism as a strategy to increase long-lasting happiness [89]. Additionally, optimism can be developed and trained so that students can focus on the positive [89,90,91]. Optimism can be incorporated along with gratitude or happiness interventions [92,93] to reduce depressive symptoms.

## 5. Conclusions

Optimism is an affective resource that influences life positively [6,7]. The present paper provides a scale that confirms the measurement of optimism from an ethnopsychological perspective. Validity, reliability and invariance were determined, as well as percentiles for the practical use of the scale. A convergent validity assessment confirmed correlations in the expected direction with variables such as positive and negative emotional states and coping. Due to the good psychometric properties obtained, the MOS can be used as a complementary scale when carrying out evaluations in different contexts, such as work, psychotherapy or educational counseling. Finally, the results provide strong psychometric evidence for its use, suggesting that the scale is an important instrument for assessing the construct of optimism and that it may benefit future research.

## Figures and Tables

**Table 1:** Descriptive statistics for total items (*n* = 848).

	M	SD	g1	g2	SW	*p*
*1. Soy positivo (a) en cualquier situación/*I am positive in any situation.	3.46	0.89	−0.10	−0.18	0.886	<0.001
*2. Trato de ver lo bueno de las cosas y esperar lo mejor/*I try to see the good in things and hope for the best.	3.57	0.93	−0.24	−0.34	0.891	<0.001
*3. Veo cosas buenas dentro de lo malo/*I see good things within the bad.	3.47	0.94	−0.10	−0.49	0.895	<0.001
*4. Tengo una visión positiva de la vida/*I have a positive outlook on life.	3.58	0.97	−0.25	−0.45	0.893	<0.001
*5. Veo las situaciones de la mejor manera posible/*I see situations in the best possible way.	3.46	0.90	−0.02	−0.29	0.884	<0.001
*6. Procuro ver el lado positivo a cualquier situación/*I try to see the positive side of any situation.	3.52	0.92	−0.09	−0.44	0.890	<0.001
*7. Espero que las cosas se den de la mejor manera/*I hope things turn out for the best.	3.82	0.89	−0.54	0.14	0.866	<0.001
*8. Trato de ver lo positivo de las situaciones desfavorables/*I try to see the positive in unfavorable situations.	3.42	0.95	−0.06	−0.38	0.895	<0.001
*9. Pienso en cosas buenas, aunque me vaya mal/*I think of good things, even if things go wrong.	3.19	0.97	0.12	−0.42	0.899	<0.001
*10. Encuentro el lado bueno en cada situación/*I find the good side in every situation.	3.39	0.89	0.05	−0.31	0.885	<0.001
*11. Mantengo un estado de ánimo entusiasta para seguir adelante/*I maintain an enthusiastic mood to move forward.	3.45	0.96	−0.16	−0.37	0.897	<0.001
*12. Actuó con entusiasmo al afrontar situaciones complicadas/*Acted with enthusiasm when facing complicated situations.	3.29	0.96	−0.05	−0.33	0.901	<0.001
*13. Soy una persona alegre ante situaciones difíciles/*I am a happy person in difficult situations.	3.19	1.00	−0.03	−0.49	0.909	<0.001
*14. Mantengo un estado emocional positivo acerca del futuro/*I maintain a positive emotional state about the future.	3.51	1.00	−0.31	−0.40	0.899	<0.001
*15. Soy optimista ante cualquier problema/*I am optimistic about any problem.	3.28	0.91	0.14	−0.36	0.888	<0.001
*16. Tengo un estado de ánimo tranquilo ante situaciones que se me hacen difíciles/*I have a calm state of mind when faced with situations that seem difficult to me.	3.30	1.00	−0.04	−0.40	0.903	<0.001
*17. Trato de ver lo bueno en todas las situaciones, antes de ver lo que falta o lo que está mal/*I try to see the good in all situations, before seeing what is missing or what is wrong.	3.20	0.95	0.05	−0.44	0.901	<0.001
*18. Mantengo un estado de felicidad que me permite ver las cosas de una manera positiva/*I maintain a state of happiness that allows me to see things in a positive way.	3.27	0.91	0.02	−0.33	0.895	<0.001
*19. Tengo un estado de ánimo feliz ante circunstancias adversas/*I have a happy state of mind in the face of adverse circumstances.	3.22	0.89	0.00	−0.25	0.893	<0.001
*20. Se cómo sacarle todo lo bueno a una situación adversa/*I know how to get all the good out of an adverse situation.	3.25	0.91	0.04	−0.35	0.894	<0.001
*21. Cuando estoy contento puedo afrontar los distintos retos que se presenten día con día/*When I am happy I can face the different challenges that arise every day.	3.83	0.89	−0.40	−0.29	0.869	<0.001
*22. Enfrento con alegría las adversidades de la vida/*I face life’s adversities with joy.	3.25	0.91	−0.02	−0.13	0.893	<0.001
*23. Tengo una visión positiva sin importar las circunstancias/*I have a positive outlook regardless of the circumstances.	3.28	0.90	0.11	−0.25	0.887	<0.001
*24. Buscó lo bueno en las situaciones complicadas/*He looked for the good in complicated situations.	3.38	0.93	−0.11	−0.37	0.897	<0.001
*25. Soy positivo (a) ante circunstancias negativas/*I am positive in the face of negative circumstances.	3.25	0.92	−0.01	−0.22	0.896	<0.001
*26. Afronto las situaciones del día a día de la mejor manera/*I face day-to-day situations in the best way.	3.45	0.87	−0.14	−0.17	0.884	<0.001
*27. Pienso en cosas positivas de la vida a pesar de las dificultades/*I think of positive things in life despite the difficulties.	3.43	0.89	−0.15	−0.21	0.890	<0.001
*28. Me mantengo optimista a pesar de los momentos difíciles/*I remain optimistic despite difficult times.	3.36	0.90	−0.13	−0.22	0.893	<0.001
*29. Pienso positivamente ante una situación complicada/*I think positively in a complicated situation.	3.31	0.89	−0.09	−0.30	0.892	<0.001
*30. Busco vías de solución positivas a una situación/*I look for positive solutions to a situation.	3.67	0.88	−0.27	−0.30	0.880	<0.001
*31. Tengo esperanza de que todo puede cambiar si te lo propones, aun sabiendo que probablemente no se logre/*I have hope that everything can change if you put your mind to it, even knowing that it probably won’t be achieved.	3.75	0.98	−0.46	−0.25	0.879	<0.001
*32. Confío en que las etapas difíciles van a terminar y vendrán tiempos mejores/*I trust that the difficult times will end and better times will come.	3.95	0.94	−0.66	−0.02	0.853	<0.001
*33. Mantengo una ventana de esperanza sin importar qué tan mal esté el panorama/*I maintain a window of hope no matter how bad the outlook is.	3.67	0.93	−0.26	−0.41	0.884	<0.001
*34. Tengo la esperanza de algo positivo para mi futuro/*I am hoping for something positive for my future.	4.02	0.95	−0.74	−0.00	0.838	<0.001
*35. Mantengo la esperanza de afrontar momentos difíciles a los que me puedo encontrar/*I remain hopeful to face difficult times that I may encounter.	3.68	0.93	−0.29	−0.40	0.884	<0.001
*36. Cuento con la fortaleza necesaria para poder afrontar momentos difíciles/*I have the necessary strength to be able to face difficult times.	3.75	0.95	−0.30	−0.51	0.877	<0.001

Note: the original Spanish version is in italics. M = mean, SD = standard deviation, g1 = skewness, g2 = kurtosis, SW = Shapiro–Wilk.

**Table 2:** Exploratory and confirmatory factor analyses of the optimism scale.

	EFA (n = 400)			CFA (n = 448)		
Item	F1	F2	F3	Affective Resources	Positive Vision	Hope
19	0.873			0.656		
13	0.873			0.713		
12	0.732			0.762		
22 *	0.689					
18 *	0.675					
16	0.674			0.547		
17 *	0.647					
25 *	0.641					
20 *	0.624					
23 *	0.608					
29 *	0.595					
24 *	0.563					
27 *	0.505					
11	0.439			0.737		
6		0.902			0.798	
5		0.843			0.734	
2		0.827			0.738	
1		0.660			0.674	
4 *		0.630				
3		0.626			0.677	
8 *		0.586				
10 *		0.504				
32			0.939			0.740
34			0.878			0.712
33			0.774			0.746
31			0.761			0.712
35			0.667			0.753
36 *			0.589			
21 *			0.548			
30 *			0.426			

Note: * items removed from the final version by means of modification indices.

**Table 3:** Fit measures of the models tested.

Model	*X* ^2^	*X* ^2^ */df*	CFI	TLI	NFI	RFI	IFI	RMSEA
EFA	2146.84	3.8	0.92	0.91	0.90	0.89	0.92	0.05
CFA (two factors)	819.304	4.8	0.93	0.93	0.92	0.91	0.93	0.06
CFA (three factors)	319.58	3.6	0.96	0.96	0.95	0.94	0.96	0.05

**Table 4:** Correlation, AVE and Fornell–Larcker criterion.

	AVE	Positive Vision	Affective Resources	Hope
Positive Vision	0.534	**0.730**		
Affective Resources	0.485	0.687 ***	**0.696**	
Hope	0.542	0.580 ***	0.557 ***	**0.736**

Note: AVE = average variance extracted, Fornell–Larcker criterion in bold and off diagonal correspond to correlations between constructs. *** *p* < 0.001.

**Table 5:** Invariance analysis of the scale according to sex.

Model	*X* ^2^	*df*	*X*^2^/*df*	CFI	RMSEA	Model Contrast	ΔCFI	ΔRMSEA
Men	399.247	167	2.39	0.957	0.058			
Women	357.048	167	2.13	0.965	0.051			
M1	2958.631	1114	2.65	0.912	0.062			
M2	2985.091	1146	2.60	0.912	0.062	M2–M1	0.000	0.000
M3	3053.121	1178	2.59	0.910	0.061	M3–M2	−0.002	−0.001
M4	3120.792	1213	2.57	0.909	0.061	M4–M3	−0.001	0.000

**Table 6:** Correlations between the optimism measure with theoretically related measures.

	Positive Vision	Affective Resources	Hope	Positive	Negative	PFE	EFD	PFD
Positive Vision	—	0.714 ***	0.604 ***	0.555 ***	−0.259 ***	0.564 ***	−0.177 ***	−0.087 *
Affective Resources	0.657 ***	—	0.580 ***	0.656 ***	−0.254 ***	0.508 ***	−0.197 ***	−0.083 *
Hope	0.555 ***	0.533 ***	—	0.472 ***	−0.148 ***	0.506 ***	−0.067	0.008 *
Positive	0.579 ***	0.618 ***	0.501 ***	—		0.491 ***	−0.241 ***	−0.151 *
Negative	−0.310 ***	−0.317 ***	−0.275 **	−0.297 ***	—	−0.273 ***	−0.389 ***	0.283 ***
PFE	0.519 ***	0.498 ***	0.530 ***	0.524 ***	−0.266 ***	—	−0.087	−0.095
EFD	−0.289 ***	−0.307 ***	−0.223 *	0.323 ***	0.420 **	−0.238 ***	—	0.211 ***
PFD	−0.101 *	−0.123 **	−0.126 **	−0.109 **	0.253 ***	−0.062	0.384 ***	—
EFE	0.318 ***	0.223 ***	0.304 ***	0.304 ***	−0.187 ***	0.402 *	−0.259 ***	0.004

Note: the data above the diagonal correspond to men, and the data below correspond to women. PFE = problem-focused engagement, EFD = emotion-focused disengagement, PFD = problem-focused disengagement, EFE = emotion-focused engagement. * *p* < 0.05, ** *p* < 0.01, *** *p* < 0.001.

**Table 7:** Optimism scale percentile scores.

	Women			Men		
Percentile	Positive Vision	Affective Resources	Hope	Positive Vision	Affective Resources	Hope
1	10	7	12	9	7	9
10	15	11	17	15	12	17
20	17	13	18	18	14	19
25	18	14	19	18	15	20
30	18	14	20	19	15	21
40	19	15	22	20	16	23
50	21	16	23	21	18	24
60	22	17	24	23	18	24
70	23	18	25	24	19	25
75	24	18	26	24	20	26
80	24	19	27	25	20	27
90	27	20	29	28	22	29
99	30	25	30	30	25	30
Mean	20.80	15.92	22.68	21.40	17.05	23.02
Median	21.00	16.00	23.00	21.00	17.00	24.00
SD	4.50	3.62	4.52	4.71	3.83	4.71

## Data Availability

All data and text material are available upon request from the first author.
